# Unveiling the Molecular Basis of Mascarpone Cheese Aroma: VOCs analysis by SPME-GC/MS and PTR-ToF-MS

**DOI:** 10.3390/molecules25051242

**Published:** 2020-03-10

**Authors:** Vittorio Capozzi, Valentina Lonzarich, Iuliia Khomenko, Luca Cappellin, Luciano Navarini, Franco Biasioli

**Affiliations:** 1 Institute of Sciences of Food Production, National Research Council (CNR), URT c/o CS-DAT, Via Michele Protano, 71121 Foggia, Italy; vittorio.capozzi@ispa.cnr.it; 2 Aromalab, illycaffè s.p.a., Area di Ricerca, Padriciano 99, 34149 Trieste, Italy; valentina.lonzarich@illy.com; 3 Department of Food Quality and Nutrition, Research and Innovation Centre, Fondazione Edmund Mach (FEM), via E. Mach 1, 38010 San Michele all’Adige, Italy; iuliia.khomenko@fmach.it (I.K.); franco.biasioli@fmach.it (F.B.); 4 Department of Chemical Sciences, University of Padua, Via F. Marzolo 1, 35131 Padova, Italy; luca.cappellin@unipd.it

**Keywords:** mascarpone cheese, dairy product, VOCs, PTR-ToF-MS, HS-SPME GC-MS, aroma, ketones, alcohols, Tiramisù, milk cream

## Abstract

Mascarpone, a soft-spread cheese, is an unripened dairy product manufactured by the thermal-acidic coagulation of milk cream. Due to the mild flavor and creamy consistency, it is a base ingredient in industrial, culinary, and homemade preparations (e.g., it is a key constituent of a widely appreciated Italian dessert ‘Tiramisù’). Probably due to this relevance as an ingredient rather than as directly consumed foodstuff, mascarpone has not been often the subject of detailed studies. To the best of our knowledge, no investigation has been carried out on the volatile compounds contributing to the mascarpone cheese aroma profile. In this study, we analyzed the Volatile Organic Compounds (VOCs) in the headspace of different commercial mascarpone cheeses by two different techniques: Headspace-Solid Phase Microextraction-Gas Chromatography-Mass Spectrometry (HS-SPME GC-MS) and Proton-Transfer Reaction-Mass Spectrometry coupled to a Time of Flight mass analyzer (PTR-ToF-MS). We coupled these two approaches due to the complementarity of the analytical potential—efficient separation and identification of the analytes on the one side (HS-SPME GC-MS), and effective, fast quantitative analysis without any sample preparation on the other (PTR-ToF-MS). A total of 27 VOCs belonging to different chemical classes (9 ketones, 5 alcohols, 4 organic acids, 3 hydrocarbons, 2 furans, 1 ester, 1 lactone, 1 aldehyde, and 1 oxime) have been identified by HS-SPME GC-MS, while PTR-ToF-MS allowed a rapid snapshot of volatile diversity confirming the aptitude to rapid noninvasive quality control and the potential in commercial sample differentiation. Ketones (2-heptanone and 2-pentanone, in particular) are the most abundant compounds in mascarpone headspace, followed by 2-propanone, 2-nonanone, 2-butanone, 1-pentanol, 2-ethyl-1-hexanol, furfural and 2-furanmethanol. The study also provides preliminary information on the differentiation of the aroma of different brands and product types.

## 1. Introduction

Mascarpone cheese is a soft-spread dairy unripened product manufactured by the thermal-acidic coagulation of milk cream [1]. Mascarpone represents an interesting cheese processing method, in which direct acidification is applied. The raw materials for its production are milk cream (containing 80% dry weight lipids, 2.8% to 6% protein) and acidifying substances (single or mixed), such as acetic, citric, tartaric, or lactic acids, vinegar or lemon juice, with a final pH ranging from 5.7 to 6.6 [2]. The cream is heated up to 85–95 °C and, while stirring, acid is added in order to force matrix coagulation [3,4]. During the intensive heating, the whey protein denatures and aggregates or sticks to the casein micelles and the fat globule membrane [3]. As a result of this reaction, whey proteins partly remain in the cheese matrix during the draining step (about 20 h), obtaining the typical texture and flavor of mascarpone cheese [3]. This typical Italian cheese was once produced domestically by the farmers of some northern regions and consumed immediately after production. Due to its traditional importance, mascarpone is included in the list of traditional agro-food products (*Prodotto Agroalimentare Tradizionale*) [5], a list of Italian traditional regional food products. More recently, it has been industrially produced to satisfy the increasing demand driven by two main sensory characteristics—the mild flavor and the creamy consistency. In fact, due to these attributes, mascarpone cheese is a base ingredient in industrial, culinary, and homemade preparations. The best example is its use in the preparation of one of the most widely appreciated Italian desserts—the Tiramisù. In spite of its popularity and its increasing economic relevance, the scientific literature does not report a characterization of Volatile Organic Compounds (VOCs) released by this peculiar dairy matrix. In order to characterize for the first time the VOCs associated with the headspace of mascarpone cheese, among various analytical techniques, we exploit the complementarity of Gas Chromatography-Mass Spectrometry (GC-MS) and Proton Transfer Reaction-Mass Spectrometry coupled to a Time of Flight mass analyzer (PTR-ToF-MS) [6]. Gas Chromatography-Mass Spectrometry (GC-MS) is the reference method in the analysis of VOCs in the field of environmental, food, flavour and fragrance, medical and forensic sciences [7]. Solid-Phase Microextraction (SPME) combined with static headspaces (HS-SPME), in particular, offers relatively high-throughput performance and does not require extended sample preparation [8]. Moreover, it is reproducible, simple, and effective, and eliminates interference compounds from the sample matrix with improvement in the selectivity of the analysis. PTR-ToF-MS uses proton transfer to induce chemical ionization of the sample headspace directly introduced into a drift tube, where volatile organic compounds can react with H_3_O^+^ ions formed in a hollow cathode ion source. The protonated particles are analyzed according to their mass/charge ratio (*m/z*) using a quadrupole or Time-of-Flight (ToF) mass analyzer and eventually detected as ion counts/second (cps) by a secondary electron multiplier or multichannel plates [9]. The outcome is a rapid (< 1 s) mass resolved fingerprint of the total volatile profile of the sample, measuring most VOCs at ultralow concentrations (a few pptv) and high mass resolution [10]. These analytical approaches are complementary. In fact, PTR-MS provides analytical information that is mostly limited to concentration and m/z ratios, i.e., sum formula, while isobar separation and compound identification needs usually the support of GC analysis [6]. PTR-MS, however, guarantees rapid and direct analysis and high sensitivity [11].

Using this integrated approach, the present study represents a first step towards the comprehension of the molecular basis of sensory perceptions associated with the consumption of mascarpone cheese and, more relevantly, of products that use mascarpone as raw material. Furthermore, within the panel of tested samples, we preliminary explored variables such as different manufacturers and delactosed mascarpone productions.

## 2. Results and Discussion

### 2.1. HS-SPME GC-MS Results

Solid-phase microextraction (SPME) is a very popular analytical extraction technique used before GC-MS headspace (HS) analysis thanks to its ease-to-use, the possibility of automation, and good sensitivity. SPME utilizes a short, thin, solid rod of fused silica coated with an absorbent/adsorbent polymer. The coated fused silica (the SPME fiber) is attached to a metal rod, and both are protected by a metal sheath that covers the fiber when not in use. SPME is particularly well suited to the analysis of dairy products being capable of extracting a broader range of analytes than most other sample preparation methods [12]. Moreover, thanks to the relatively low temperatures and short times at which headspace SPME extraction is performed, the risks to induce thermal artifacts are extremely low if compared with other techniques such as simultaneous distillation-extraction (SDE) [13].

Methods developed for the analysis of organic compounds from aqueous samples by SPME coupled to GC have been used to analyze VOCs in fresh and ripened dairy productions [8,14]. A wide range of fibers with varying affinities for specific classes of volatile organic compounds is available. After a preliminary screening of seven different types of SPME fibers (100 µm PDMS (polydimethylsiloxane), 60 µm PEG (Carbowax-Polyethylene Glycol), 85 µm PA (Polyacrylate), 75 µm CAR/PDMS (Carboxen/Polydimethylsiloxane), 85 µm CAR/PDMS, 50/30 µm DVB/CAR/PDMS (Divinylbenzene/Carboxen/Polydimethylsiloxane), and 65 µm PDMS/DVB (Polydimethylsiloxane/Divinylbenzene)) 75 µm CAR/PDMS was chosen, because it provides the higher number of extracted volatiles. This fiber has been suggested to work particularly well for the analysis of volatiles in dairy products [12]. Extraction conditions have also been preliminarily explored by checking the effect of different times (10 min up to 4 h) and temperature (40 and 60 °C). A good compromise between the multiplicity of extracted volatiles and peak intensity was found by headspace exposing the fiber for 60 min at 60 °C and this experimental condition was selected for the present characterization.

A total of 27 compounds belonging to different chemical classes (nine ketones, five alcohols, four acids, three hydrocarbons, two furans, one ester, one lactone, one aldehyde, and one oxime) have been identified. Ketones, which might induce fruity and floral sensory notes, are common constituents of most dairy products [15,16,17] and by far the most important class of compounds contributing to the mascarpone cheese aroma. In particular, 5 different ketones (2-heptanone > 2-pentanone > 2-propanone > 2-nonanone ≈ 2-butanone) represent almost 75–80% of the sample headspace. The compounds 2-heptanone and 2-pentanone characterized by odor descriptors including sweet, fruity, orange peel, and herbaceous [15] are the dominating volatile organic compounds in all samples. Several alcohols have been detected, but differently from ketones, are not present in all samples—1-pentanol and 2-ethyl-1-hexanol, both common primary alcohols detected in dairy products [15], are ubiquitous, and ethanol and 1,2 propandiol have been detected only in one sample (Manufacturer B), suggesting a possible technological origin. Other minor compounds, including short- and moderate-chain even-numbered fatty acids (C_4_–C_12_), ethyl acetate, δ-hexalactone, toluene, benzaldehyde and methoxyphenyl oxime have been already found in cheese products [16,18,19]. The two hydrocarbons 2,4-dimethylheptene and 2,2,4,6,6-pentamethylheptane have been detected only in one sample (Manufacturer A) and the latter has been detected in the volatile fraction of butter [20]. Furfural and 2-furanmethanol, identified in all mascarpone cheese samples, have been found to contribute to the nutty and roasted aroma of Parmigiano-Reggiano cheese [21].

**Figure 1:**
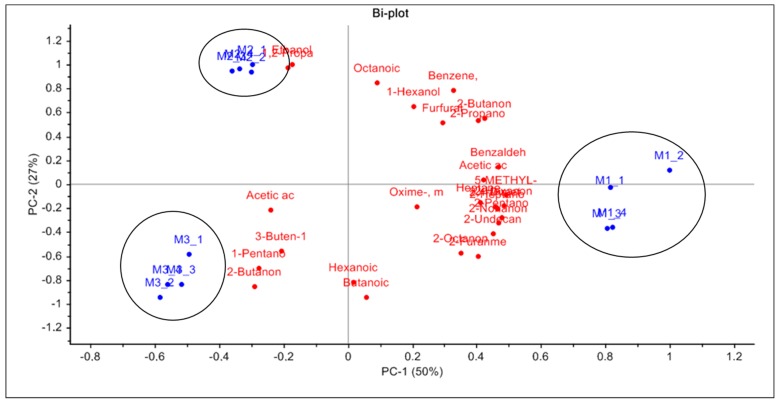
Principal Component Analysis (PCA) biplot of the 3 different commercial samples of mascarpone (M1, M2, and M3). For each sample, the mean (n = 4) is represented by the sample name. Score plot was given by the Volatile Organic Compound (VOC) content for each sample and loading plot of the single volatile organic compounds. The codes correspond to the samples indicated in Table 1.

In order to provide a general overview of volatile composition of three different samples (M1-M3) of Mascarpone cheese analyzed by HS-SPME GC-MS, we performed multivariate data analysis using Principal Component Analysis (PCA), reporting the graphical result in Figure 1.

In the figure, it is possible to observe the samples (scores) and variables (loadings) plots related to the first two principal components, which (cumulated) explain the 77% of the total variance (PC1, 50.0%; PC2, 27.0%) associated with the data set. A clear separation of mascarpone cheese M1 from the other samples is observable along the PC1. PC2 explains the parting between M2 and M3 mascarpone samples. The replicates belong to the same commercial mascarpone batch that is well-clustered together, while is possible to highlight a clear separation of the three samples on the biplot. Observing the loadings (i.e., the involvement of the single volatiles), it is possible to have an idea of the different influence of the diverse volatiles in justifying variance observed trends.

### 2.2. PTR-ToF-MS Results and Comparison with HS-SPME GC-MS Findings

As other Direct-Injection Mass Spectrometric (DIMS) technologies, PTR-MS finds application in many sectors, from environmental sciences to food chemistry, and from biological studies to medical applications. With this regard, we recently described a tailored system, that found application in this study, achieved connecting PTR-ToF-MS with an automated sampler, and associated custom-made data analysis applications that improve the versatility of the analytical approach in the determination of VOCs in association with i) huge numbers of samples, ii) bioprocesses monitoring, and iii) high numbers of variables to be considered [11].

PTR-MS has been already exploited to study VOCs associated to dairy products such as mozzarella cheese [22], Grana Padano, Parmigiano Reggiano, and Grana Trentino cheeses [23], liquid whey [24], butter and butter oils (by means of quadrupole-based PTR-MS analyses, sensory analyses and classical chemical analyses) [25], milk and whey powders [26], anhydrous milk fat [26], and fermented milk-based beverages (yogurt and kefir) [27,28].

**Figure 2:**
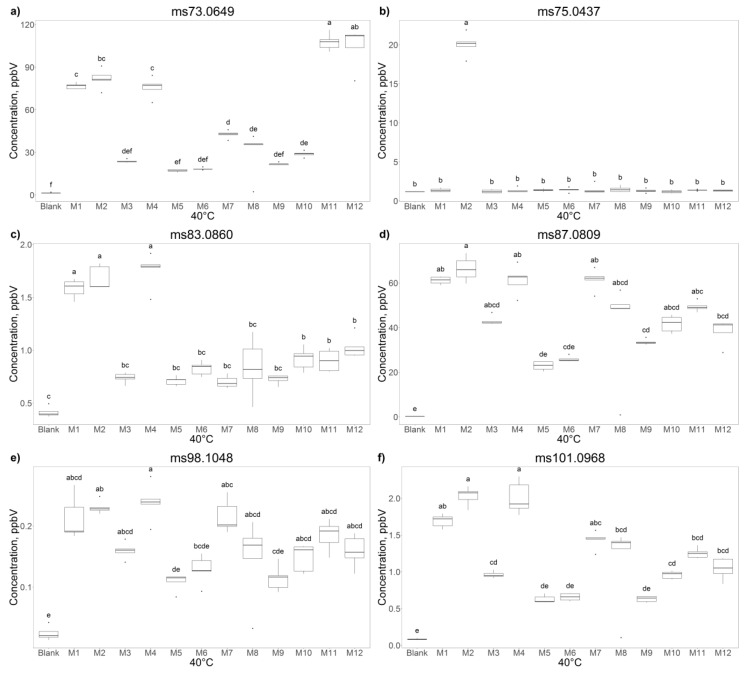
The boxplots indicated by letters (**a**–**f**) represent selected volatiles found in association with the different commercial mascarpone samples such as m/z 73.0649—C_4_H_9_O^+^—t.i. 2-Butanone, 75.0437—C_3_H_7_O_2_^+^—t.i. Methyl acetate, 83.0860—C_6_H_11_^+^—t.i. fragment of Hexanal/Hexenol, 87.0809—C_5_H_11_O^+^—t.i. 2-Pentanone/3-Buten-1-ol, 3-methyl-, 98.1048—isotope of C_7_H_13_^+^—t.i. Heptanal, 101.0968—C_6_H_13_O^+^—t.i. 2-Hexanone. Different letters indicate a significant difference between different samples (*p* < 0.05, one-way ANOVA, Tukey HSD).

All samples included in this study have been analyzed by PTR-ToF-MS. A total of 411 mass peaks were detected and extracted. Upon comparison with the blanks, 92 peaks were kept that are significantly different between various manufacturers (*p* < 0.01 with Bonferroni correction) and tentatively identified on the basis of exact mass, isotopic ration, and literature [29]. PTR-MS allowed the detection and characterization of a larger number of VOCs/VOC fragments, which was larger than the number of volatiles identified by GC. For the PTR analysis, all vials were incubated alternatively at 40 °C or at 60 °C (data not shown) for 30 min before PTR-MS analysis. The last one was the temperature at which good results were obtained by HS-SPME GC-MS. However, with PTR, even at 40 °C, the analysis was successfully performed and results were reliable. For this reason, we report the data performed at 40 °C, a temperature closer to the real mascarpone cheese testing conditions. One-way ANOVA followed by Tukey HSD test was carried out to compare and underline significant differences among the assessed mascarpone samples. For each peak, we obtained the concentration of the corresponding VOC ion in the headspace of all explored samples. Boxplots reported in Figure 2 illustrate the observed trends for 6 ions among the tested samples, as illustrative cases. In detail, the figure proposes the behaviors corresponding to the peaks at *m/z* 73.065 (tentatively identified as 2-butanone), *m/z* 75.044 (tentatively identified as propionic acid), m/z 83.086 (tentatively identified as hexanol fragment), *m/z* 87.080 (tentatively identified as 2-pentanone/isoprenol), *m/z* 98.105, and *m/z* 101.096 (tentatively identified as 2-hexanone). The intensity corresponding to the mass peak *m/z* 73.065 reaches the highest values in the delactosed samples produced by Manufacturer C, while the standard productions belonging to the same manufacturer registered the lowest values (as all mascarpone batches of Producer M) (Figure 2a). Samples from Manufacturers A and B present intermediate intensities for this peak (Figure 2a). In accordance with these results, 2-butanone was found to be variable in different types of whey [30]. In only the M2 batch did we detected a relevant intensity for the mass peak *m/z* 75.044 (Figure 2b), tentatively identified as propionic acid, a compound that can be responsible for a dairy taste/odor with a pronounced fruity lift [31].

The mass peak *m/z* 83.086 has been found with pronounced intensities in the samples produced by the Manufacturers A and B (Figure 2c). Hexanal was included among the high-content compounds identified in samples belonging to dairy products [32] and described as having a fatty, green, grassy, powerful, penetrating characteristic fruity odor and taste [31]. A similar trend can be underlined for the intensities of mass peak *m/z* 101.096 (Figure 2f). Finally, a considerable variability can be highlighted for the intensities corresponding to the mass peaks m/z 87.080 and 98.105 (Figure 2d,e).

Other than this kind of punctual analysis, PTR analysis offers also the opportunity to depict a global analysis of molecular fingerprinting associated with the headspaces of the different samples. Considering that the present work deals with an integrated analytical approach, we propose a PTR data set selected in light of the comparison with GC data. In fact, we defined a new subset of the PTR-ToF-MS data including only the mass peaks that were found also using the HS-SPME GC-MS technique. As a result, we have a new matrix (Table 2) of twenty peaks corresponding to the masses of protonated molecular ions of compounds such as acetic acid (sour pungent, cider vinegar, slightly malty with a brown nuance; naturally occurring in various dairy products, it has a role in butter and cheese flavors), acetoin (acidic, sour, cheesy, dairy, creamy with a fruity nuance; normally occurs in butter, milk, and cheeses), acetone (characteristic aromatic odor, pungent, somewhat sweet taste; naturally occurring in fermented dairy products), ethanol (slight, characteristic odor and a burning taste; naturally occurring in blue cheese, cheddar cheese, Swiss cheese), furfural (characteristic penetrating odor typical of cyclic aldehydes; naturally occurring in cheeses), hexanoic acid (sickening, sweaty, rancid, sour, sharp, pungent, cheesy, fatty, unpleasant odor reminiscent of copra oil; naturally occurring in cheeses, butter, milk), and octanoic acid (mildly unpleasant odor and a burning, rancid taste, also reported as having a faint, fruity-acid odor and slightly sour taste; natural component of butter fat, occurring in cheeses) [31,33].

**Figure 3:**
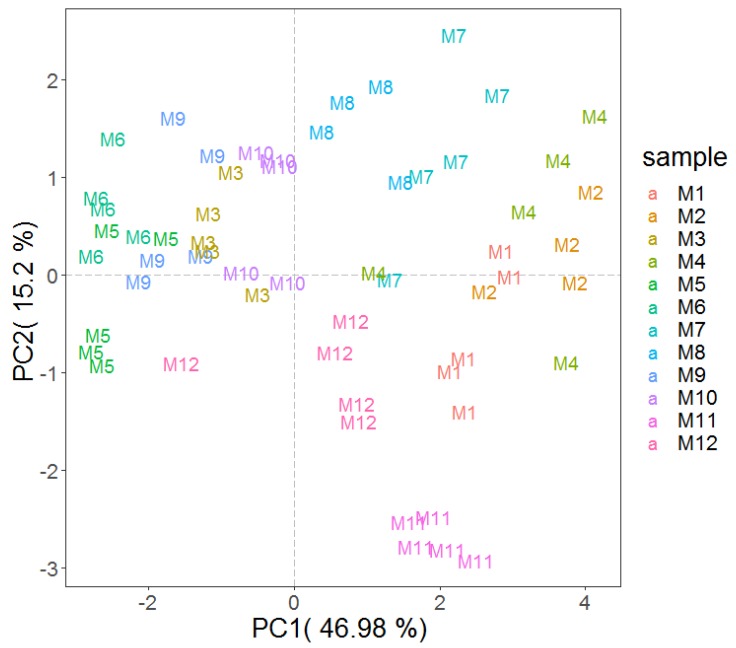
Analysis of mascarpone VOCs profile assessed by PTR-ToF-MS. Plot depicts the VOC profile distribution of the twelve Mascarpone over the PCA score plot defined by the first two principal components. The codes correspond to the samples indicated in Table 1.

Statistical tests were performed on the new matrix in an attempt at understanding the impact of these VOCs on the characterization of the different mascarpone cheese samples. The results obtained for the twelve experimental modes were visualized by means of principal component analysis (PCA), with each point representing a distinct sample (Figure 3), maximizing explained variability in two dimensions.

Separation among Mascarpone samples according to the first two components accounted for about 62% of the total variance. It is possible to highlight how the replicates belonging to the same sample generally clustered together. In addition, a good separation among the different samples is also depicted. Considering all variables connoting the panel of different mascarpone cheese analyzed, it is mandatory to underline that the studied diversity in terms of different producers and classic versus delactosed was not selected in order to delve into the effect of these parameters. In fact, it was just a heterogeneous panel selected in order to provide a broad description of the overall VOCs associated with this traditional dairy production. However, it is possible to foresee some preliminary differences, such as clear groups among mascarpone cheese samples belonging to the same manufacturer and a general (more or less pronounced) separation between classic and delactosed samples within the same producer (Figure 3). These pieces of evidence suggest the need for further studies with tailored sampling in order to test the potential of a PTR-based approach as a discriminatory tool to monitor these variables. Considering the sensory changes among mascarpone cheese samples, our study confirmed the presence of a diversification comparing different batches and different producers already described in terms of spreadability [34]. In fact, Cattaneo et al. [34], studying eighteen batches from six different manufacturers, noticed differences in four viscometric parameters they selected to assess changes of rheological aptitude of mascarpone cheeses. This sensory variability calls attention to the need for versatile tools for the industrial quality control also in the case of mascarpone cheese, a topic of generally significant interest in the food industry [35,36].

In Table 3, it is possible to delve into the results for a more representative number of mass peaks, underlining significant differences among concentrations reported for 22 protonated ions out of the 92 selected after comparison with the blanks. From this analysis, it is possible to notice how the trends for selected mass peak intensities follow a certain producer-dependent behavior. It is also clear how the probabilities to find selected mass peaks associated to given experimental variables considerably increase using the PTR-based technique, due to the potential of an untargeted approach. The opportunity to have a wide (untargeted analysis) and fast (rapid time of analysis without any sample preparation/extraction/destruction) view of the VOCs associated with mascarpone headspaces confirmed the aptitude of this analytical approach to allow rapid noninvasive quality control for the food industry (e.g., [37,38]), already explored in the dairy industry but on other matrices (e.g., [25,26]). An approach that can i) simplify the selection of mascarpone as an ingredient in the food industry and ii) boost the quality improvements in the production of this fresh cheese.

This panel of 22 peaks includes only 9 masses detected also by the GC analysis, thus providing a broader overview of the diversity among samples associated with VOCs content. Comparing these findings with a recent PTR headspace analysis of other dairy product of industrial interest (milk powder, whey powder and anhydrous milk fat), the mass peaks 47.049, 63.026, 73.065, 87.081, 89.060, 101.097, 115.113, 143.145 seem to be peculiar of mascarpone headspace [26], indicating a potential role of the corresponding volatiles in shaping perceptions associated to Mascarpone consumption. Additionally, on the other hand, we found variable trends in mass peaks already detected in association with the headspaces of skim milk powder (43.018, 61.029, 87.044, 97.102), whole milk powder (41.039, 43.018, 45.033, 55.054, 61.029, 71.086, 75.044, 83.086, 87.044), whey powder (43.018, 59.049, 61.029, 75.044), and anhydrous milk fat (43.018, 43.054, 57.070, 69.070) [26]. This partial and specific overlapping, in terms of volatiles content, with the headspaces of other dairy ingredients/products, can be probably of help in the understanding of the unique sensory properties of mascarpone matrix.

**Figure 4:**
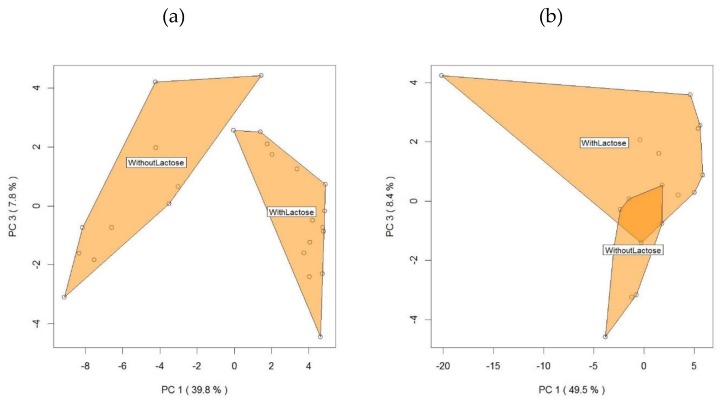
Analysis of mascarpone VOCs profile assessed by PTR-ToF-MS for the Manufacturers C (**a**) and M (**b**) plotted by the first and the third principal components. The labels and the selected areas indicate the separation between samples with or without lactose.

Finally, in order to provide more complete information about the preliminary potential that arises from the PTR data in terms of separation of delactosed products, we propose two PCA representations, analyzing samples with or without lactose for the Manufacturers C and M, respectively (Figure 4).

Figure 4a (Manufacturer C) and 4b (Manufacturer M) show that mascarpone samples classic and delactosed in this subset (different producers) are separated along the first and third PC (explaining 47.6% and 57.9% of the total variance, respectively). Even if preliminary, these results confirm the potential of PTR-TOF-MS analysis for the quality evaluation of lactose-free dairy products. In fact, recently, this analytical approach found application to monitor VOC variability in ultrahigh temperature lactose-free milk samples (assessing the impact of storage time and the of the use of different lactase preparations) [39]

## 3. Materials and Methods

### 3.1. Sample Selection and Preparation

A total of 12 different mascarpone batches were studied in this project that are listed in Table 1. The corresponding chemicophysical characteristics are reported in Table S1.

We obtained the samples from different local markets and stored them at 4 °C. The samples represent different manufacturers, all analyzed within the expiration date, and both plain and delactosed Mascarpone.

### 3.2. HS-SPME GC-MS Measurements

Aliquots of 8 mL of sample were placed in a 20 mL vials that were immediately sealed with a silicone rubber Teflon cap and crimped with aluminium seal. Then samples were heated at 60 °C and kept at the same temperature for 30 min while a polydimethylsiloxane/divinylbenzene SPME fibre (Supelco, Bellefonte, PA, USA) was exposed to the headspace over the surface of each sample in order to collect the compounds in the vapour phase. The exposure time was optimized in preliminary experimental trials. The SPME coating containing the headspace volatile compounds was inserted into the GC injection port and then thermally desorbed at 250 °C for 10 min in a 6890 GC (Agilent Technologies, Santa Clara, CA, United States). Compounds were eluted by a He gas flow of 1,4 mL/min in split mode (split 1:4) and separated using a 60 m Varian FactorFour WAXms capillary column (film thickness 0.25 mm, 0.25 mm internal diameter) (Varian, Middelburg, The Netherlands). The oven temperature, initially set to 35 °C, was increased to 210 °C at 4 °C/min, then to 240 °C at a rate of 20 °C/min, and then this final temperature was held for 5 min. The mass spectrometer was set to electron ionization mode (MS-EI) generated at 70 eV, and mass spectra were collected in full scan mode, collecting ions from 39 to 250 *m/z*. The volatile compounds studied were identified by comparing their mass spectra and their retention times to those of reference standards analyzed at the same conditions and by comparison with spectra recorded in the Wiley 6 N mass spectral library (Wiley, Hoboken, NJ, USA) and, when needed, to literature references. Due to the lengthy HS-SPME GC/MS analysis, only four samples have been analysed by this method. For each sample, four replicates were analyzed.

### 3.3. PTR-ToF-MS Measurements

A commercial PTR-ToF-MS 8000 instrument (Ionicon Analytik GmbH, Innsbruck, Austria) was used for the headspace measurements. The instrumental conditions in the drift tube were as following—drift voltage 550 V, drift temperature 110 °C, drift pressure 2.30 mbar affording an E/N value of 140 Townsend (1 Td = 10^−17^ V.cm^2^). Sampling was performed with a flow rate of 40 sccm. The mass resolution (m/Δm) was at least 3800. Measurements were performed in an automated way by using a multipurpose GC automatic sampler (Gerstel GmbH, Mulheim am Ruhr, Germany) as previously described [11]. The measurement order, both samples and replicates, was randomized to avoid memory effects. All vials were incubated at 40 °C for 30 min before PTR-MS analysis. Each sample was measured for 30 s, at an acquisition rate of 1 spectrum per second with an overall throughput of one sample every 5 min. The experiment was repeated at 60 °C, the temperature at which HS-SPME GC-MS provided better results. The entire experiment was repeated three times and empty vials, containing lab air, were measured together with the sample set and considered as “blanks”. Data processing of PTR-ToF-MS spectra included dead time correction, external calibration and peak extraction steps performed according to a procedure described elsewhere [40]. The baseline of the mass spectra was removed after averaging the whole measurement and peak detection and peak area extraction was performed by using modified Gaussian to fit the data [41]. To determine the concentrations of volatile compounds in ppbv (part per billion by volume) the formulas described by Lindinger et al. were used by assuming a constant reaction rate coefficient (k*_R_*=2 × 10^−9^ cm^3^/s) for H_3_O^+^ as primary ion [42].

### 3.4. Statistical Analyses

Data exploration was based on Principal Component Analysis (PCA) of centered and scaled data. Analysis of variance (ANOVA) with Bonferroni correction was performed for selection of mass peaks in the sample headspace which are significantly higher than blanks. After this step, one-way ANOVA followed by Tukey’s HSD (*p* < 0.05) was applied to evaluate the significant differences among mascarpone samples. All analyses were performed with core functions of R programming language (R Development Core Team, R Foundation for Statistical Computing, Vienna, Austria, 2014) and its external packages (ChemometricsWithR, DiscriMiner, prospectr). In some cases, in order to interpret the results of the experiment, the entire dataset was divided into smaller subsets based on different criteria (e.g., producer, lactose content).

## 4. Conclusions

Using two complementary analytical approaches, Headspace-Solid Phase Microextraction-Gas Chromatography-Mass Spectrometry (HS-SPME GC-MS) and Proton-Transfer Reaction-Mass Spectrometry coupled to a Time of Flight mass analyzer (PTR-ToF-MS), the present work provides a first description of Volatile Organic Compounds (VOCs). In addition, we underline the differences in VOC content susceptible to characterize the aroma of different brands and product types (classic and lactose-free). On the whole, the dominance of volatiles generally associated to floral, fruity, sweet, and nutty notes might contribute to explain the delicate sensory impression perceived by smelling this fresh dairy product. Unfortunately, the aroma profile of the present investigation cannot be discussed in light of previous literature that is, as mentioned, very scarce. Considering the wide number of products that use mascarpone as raw material, such as the popular Tiramisù and coffee mascarpone cream, this study provides information to design future studies conceived to assess the contribution of this unripened cheese to the sensory characteristics of final products.

## Figures and Tables

**Table 1:** List of commercial ‘Mascarpone’ samples analyzed in the present study. All samples (M1–M12) were investigated by PTR-ToF-MS analysis. Underlined samples (M1–M3) were evaluated also by HS-SPME GC-MS.

Sample	Claimed Characteristics	Manufacturer
**M1**	Mascarpone	A
**M2**	Mascarpone	B
**M3**	Mascarpone	C
**M4**	Mascarpone	B
**M5**	Mascarpone	C
**M6**	Mascarpone	C
**M7**	Mascarpone	M
**M8**	Mascarpone	M
**M9**	Mascarpone without lactose	M
**M10**	Mascarpone without lactose	M
**M11**	Mascarpone without lactose	C
**M12**	Mascarpone without lactose	C

**Table 2:** Volatile compounds detected by both Proton Transfer Reaction-Mass Spectrometry coupled to a Time of Flight mass analyzer (PTR-ToF-MS) and SPME/GC-MS in association with mascarpone samples.

Compound	Chemical Class	Protonated Ion	
		*m/z*	Sum Formula
Ethanol	Alcohols	47.049	C_2_H_7_O^+^
2-Propanone	Ketones	59.049	C_3_H_7_O^+^
Acetic acid	Organic acids	61.028	C_2_H_5_O_2_^+^
2-Butanone	Ketones	73.065	C_4_H_9_O^+^
1,2-Propanediol = Propylene glycol	Alcohols	77.060	C_3_H_9_O_2_^+^
2-Pentanone/3-Buten-1-ol, 3-methyl-	Ketones/Alcohols	87.080	C_5_H_11_O^+^
2-Butanone, 3-hydroxy- (B) / Butanoic acid/Acetic acid ethyl ester	Ketones/Organic acids/Esters	89.060	C_4_H_9_O_2_^+^
Toluene	Hydrocarbons	93.070	C_7_H_9_^+^
Furfural	Furans	97.028	C_5_H_5_O_2_^+^
2-Hexanone	Ketones	101.096	C_6_H_13_O^+^
Benzaldehyde	Aldehyde	107.049	C_7_H_7_O^+^
5-Methyl-delta-valerolactone	Lactones	115.075	C_6_H_11_O_2_^+^
2-Heptanone	Ketones	115.112	C_7_H_15_O^+^
Hexanoic acid	Organic acids	117.091	C_6_H_13_O_2_^+^
2,4-Dimethyl-1-heptene	Hydrocarbons	127.148	C_9_H_19_^+^
2-Octanone	Ketones	129.127	C_8_H_17_O^+^
1-Hexanol, 2-ethyl-	Alcohols	131.143	C_8_H_19_O^+^
2-Nonanone	Ketones	143.143	C_9_H_19_O^+^
Octanoic acid	Organic acids	145.122	C_8_H_17_O_2_^+^
Oxime-, methoxy-phenyl-	Oxime	152.071	C_8_H_10_NO_2_^+^
2-Undecanone	Ketones	171.174	C_11_H_23_O^+^
Heptane, 2,2,4,6,6-pentamethyl	Hydrocarbons	171.211	C_12_H_27_^+^

**Table 3:** Organic compounds associated to mascarpone headspace detected by PTR-ToF-MS. Black color indicates compounds identified also by SPME/GC-MS. For each compound, different letters indicate a significant difference between different samples according to ANOVA and Tukey HSD (*p* < 0.05). The codes correspond to the samples indicated in Table 1. In the parenthesis, the different producers.

MM	TM	SF	M1 (A)	M2 (B)	M3 (C)	M4 (B)	M5 (C)	*p*-Value
41.039	41.039	C_3_H_5_^+^	21.2 ± 0.9 ^b^	27 ± 3 ^c^	11.4 ± 0.6 ^a^	21 ± 1 ^b^	10 ± 1 ^a^	1 × 10^−13^
43.018	43.018	C_2_H_3_O^+^	30.0 ± 0.7 ^b^	44 ± 3 ^c^	24 ± 2 ^a^	33 ± 2 ^b^	24 ± 5 ^a^	3 × 10^−9^
43.054	43.054	C_3_H_7_^+^	11.5 ± 0.6 ^c^	16 ± 1 ^d^	3.5 ± 0.5 ^a^	6.7 ± 0.4 ^b^	3.1 ± 0.4 ^a^	4 × 10^−17^
45.033	45.033	C_2_H_5_O^+^	113 ± 6 ^b^	175 ± 12 ^c^	81 ± 3 ^a^	114 ± 10 ^b^	88 ± 23 ^a^	2 × 10^−9^
47.049	47.049	C_2_H_7_O^+^	8 ± 3 ^a^	52 ± 39 ^c^	10 ± 8 ^a^	16 ± 1 ^ab^	44 ± 5 ^bc^	2 × 10^−3^
55.054	55.054	C_4_H_7_^+^	13.3 ± 0.4 ^c^	14.9 ± 0.8 ^d^	8.1 ± 0.3 ^b^	15 ± 1 ^d^	6.0 ± 0.4 ^a^	7 × 10^−16^
57.070	57.070	C_4_H_9_^+^	6.0 ± 0.1 ^c^	5.6 ± 0.6 ^c^	3.9 ± 0.2 ^b^	12 ± 1 ^d^	2.7 ± 0.1 ^a^	7 × 10^−17^
59.049	59.049	C_3_H_7_O^+^	1062 ± 35 ^c^	976 ± 72 ^c^	563 ± 29 ^b^	1230 ± 116 ^d^	355 ± 26 ^a^	9 × 10^−15^
61.029	61.028	C_2_H_5_O_2_^+^	10 ± 3 ^a^	26 ± 6 ^b^	18 ± 5 ^ab^	11 ± 2 ^a^	24 ± 10 ^b^	4 × 10^−4^
63.026	63.026	C_2_H_7_S^+^	15.1 ± 0.3 ^c^	16 ± 1 ^c^	7.2 ± 0.4 ^b^	20 ± 2 ^d^	3.3 ± 0.5 ^a^	4 × 10^−16^
69.070	69.07	C_5_H_9_^+^	6.4 ± 0.2 ^a^	8.3 ± 0.6 ^b^	6.0 ± 0.4 ^a^	9.0 ± 0.8 ^b^	6.2 ± 0.6 ^a^	2 × 10^−8^
71.086	71.086	C_5_H_11_^+^	1.2 ± 0.1 ^ab^	1.7 ± 0.8 ^b^	0.7 ± 0.1 ^a^	1.3 ± 0.1 ^ab^	0.72 ± 0.05 ^a^	1 × 10^−3^
73.065	73.065	C_4_H_9_O^+^	77 ± 2 ^b^	82 ± 7 ^b^	24 ± 1 ^a^	76 ± 7 ^b^	17.2 ± 0.9 ^a^	1 × 10^−16^
75.044	75.044	C_3_H_7_O_2_^+^	1.4 ± 0.2 ^a^	20 ± 1 ^b^	1.2 ± 0.2 ^a^	1.4 ± 0.3 ^a^	1.4 ± 0.2 ^a^	1 × 10^−21^
83.086	83.086	C_6_H_11_^+^	1.6 ± 0.1 ^b^	1.7 ± 0.1 ^b^	0.7 ± 0.0 ^a^	1.8 ± 0.2 ^b^	0.71 ± 0.04 ^a^	4 × 10^−14^
87.044	87.044	C_4_H_7_O_2_^+^	3.7 ± 0.5 ^bc^	3.8 ± 0.9 ^bc^	3.0 ± 0.4 ^ab^	4.3 ± 0.4 ^c^	2.0 ± 0.3 ^a^	2 × 10^−5^
87.081	87.08	C_5_H_11_O^+^	61 ± 2 ^c^	66 ± 5 ^c^	43 ± 2 ^b^	61±6^c^	23 ± 2 ^a^	6 × 10^−13^
89.060	89.06	C_4_H_9_O_2_^+^	2.2 ± 0.6 ^a^	5.2 ± 0.6 ^c^	2.9 ± 0.3 ^ab^	2±1^ab^	3.5 ± 0.3 ^b^	2 × 10^−6^
97.102	97.101	C_7_H_13_^+^	2.3 ± 0.1 ^c^	2.7 ± 0.2 ^d^	1.8 ± 0.0 ^b^	2.6±0.1^d^	1.1 ± 0.1 ^a^	4 × 10^−14^
101.097	101.096	C_7_H_7_O^+^	1.7 ± 0.1 ^c^	2.0 ± 0.1 ^d^	1.0 ± 0.0 ^b^	2.0±0.2^d^	0.6 ± 0.1 ^a^	5 × 10^−14^
115.113	115.112	C_7_H_15_O^+^	28 ± 1 ^c^	30 ± 2 ^c^	20.5 ± 0.6 ^b^	30±2^c^	12 ± 1 ^a^	7 × 10^−14^
143.145	143.143	C_9_H_19_O^+^	2.1 ± 0.1 ^c^	2.5 ± 0.2 ^d^	1.7 ± 0.1 ^b^	2.4±0.2^d^	1.1 ± 0.1 ^a^	2 × 10^−12^

## Supplementary Materials

The following are available online, Table S1: Monitored chemico-physical characteristics for the list of ‘Mascarpone’ samples analyzed in the present study.
