# Supplementary material for: Unveiling the Molecular Basis of Mascarpone Cheese Aroma: VOCs analysis by SPME-GC/MS and PTR-ToF-MS
Source: Molecules. 2020 Mar 10;25(5):1242. doi: 10.3390/molecules25051242 (PMC7179404; doi:10.3390/molecules25051242)
Supplement: Supplementary file 1 [file molecules-25-01242-s001.pdf]

molecules

MDPI

Table S1. Monitored chemico-physical characteristics for the list of 'Mascarpone' samples analyzed in the present study.

Sample	Physico-chemical characteristic				
	**Fat (%)**	**Sat. fat (%)**	**Carb. (%)**	**Prot. (%)**	**Sodium (g)**
**M1**	47.7	32.5	2.8	4.7	0.050
**M2**	42.0	28.8	2.5	3.6	0.100
**M3**	41.0	28.3	4.8	4.8	0.033
**M4**	42.0	28.8	2.5	3.6	0.100
**M5**	41.0	28.3	4.8	4.8	0.033
**M6**	41.0	28.3	4.8	4.8	0.033
**M7**	42.0	30.4	4.9	3.6	0.130
**M8**	42.0	30.4	4.9	3.6	0.130
**M9**	42.0	30.1	4.8	3.7	0.130
**M10**	42.0	30.1	4.8	3.7	0.130
**M11**	41.0	28.5	3.3	3.7	0.100
**M12**	41.0	28.5	3.3	3.7	0.100
